# Comparison of iASSIST Navigation System with Conventional Techniques in Total Knee Arthroplasty: A Systematic Review and Meta‐Analysis of Radiographic and Clinical Outcomes

**DOI:** 10.1111/os.12550

**Published:** 2019-11-22

**Authors:** Jun‐tan Li, Xiang Gao, Xu Li

**Affiliations:** ^1^ Department of Orthopaedics The First Hospital of China Medical University Shenyang China

**Keywords:** Handheld navigation, Portable navigation, Surgical technique, Systematic review, Total knee arthroplasty

## Abstract

The iASSIST navigation system is a handheld accelerometer‐based navigation system that has been applied in clinical practice in recent five years. This meta‐analysis aimed to compare the radiographic and clinical outcomes of iASSIST navigation with conventional surgical techniques for patients undergoing total knee arthroplasty (TKA) and to compare the surgery time between an iASSIST group and a conventional treatment group. This systematic review and meta‐analysis included all comparative prospective and retrospective studies published in Pubmed, Embase, the Cochrane Central Register of Controlled Trials, the Web of Science and the CNKI databases over the past 20 years. Inclusion criteria were studies that compared the iASSIST navigation system with conventional TKA. The primary outcomes were mechanical axis (MA) and outliers, which means postoperative MA varus or valgus of more than 3°. Secondary outcomes were coronal femoral angle (CFA) and coronal tibial angle (CTA). Knee Society Score (KSS) was used to evaluate functional outcome. The Newcastle–Ottawa Scale (NOS) was used to assess the methodological quality of included studies. Eight studies involving 558 knees were included in this meta‐analysis. Of these, 275 patients used the iASSIST navigation system and 283 used conventional surgical techniques. A total of 5 studies were considered high quality and the other 3 were considered to be of moderate quality. The occurrence of malalignment of >3° in the iASSIST group was 13.3%, compared with 29.04% in the conventional group. Postoperative MA of the iASSIST group was significantly better than that of the conventional group (*I* ^2^ = 19%, *OR* = −0.92, 95% *CI* = −1.09 to −0.75, *P* < 0.00001). The iASSIST navigation system provided significantly increased accuracy in the coronal femoral angle (*I* ^2^ = 79%, *OR* = −0.88, 95% *CI* = −1.21 to −0.54, *P* < 0.00001) and the coronal tibial angle (*I* ^2^ = 34%, *OR* = 0.39, 95% *CI* = −0.48 to −0.30, *P* < 0.00001) compared with conventional techniques. However, the duration of surgery using the iASSIST procedure was longer and there was no significant difference in the short‐term KSS in the iASSIST group compared with the conventional group. We found that when pooling the data of included studies, the number of outliers was fewer in the iASSIST group, and compared with conventional TKA techniques, the iASSIST system significantly improved the accuracy of lower limb alignment but the duration of surgery was prolonged in addition to there being no apparent advantage in terms of short‐term functional score.

## Introduction

As the incidence of knee osteoarthritis continues to increase, the number of patients requiring total knee arthroplasty (TKA) has risen^1^. TKA is regarded as an effective treatment for end‐stage osteoarthritis and rheumatic arthritis^2^. However, 20% of patients who undergo TKA are not satisfied with the results^3^, ^4^. The success of surgery depends on many factors, including soft tissue balancing, design of prostheses, and medical complications. Furthermore, surgical factors are critically important, such as the skill and experience of the surgeon, the precision of prosthesis implantation, and the duration of surgery.

Incorrect positioning of the prosthesis may result in unacceptable tibiofemoral tracking that could bring about additional stress at the loaded surfaces of the prosthesis leading to accelerated wear and component loosening^5^. Jeffery *et al*.^6^ suggest that aseptic loosening of implants in patients with alignment exceeding 3° of varus or valgus occurs at a rate of 24% compared with a rate of only 3% in patients whose alignment is within 3° of the neutral mechanical axis (MA). Malalignment may also increase wear on polyethylene tibial bearings^7^. Several studies have shown that postoperative MA of less than 3° may reduce the risk of future TKA failure^8^, ^9^, ^10^, ^11^, ^12^, ^13^. Thus, outliers of MA were defined as being outside a 3° divergence from the MA^14^.

An intramedullary guide is used for distal femoral resection and an extramedullary or intramedullary jig for tibial resection in conventional TKA surgery. This technique relies heavily on bone markers and the surgeonʼs experience, which have limited accuracy in terms of MA reconstruction and prosthesis implantation. Because of the relative stability of the osseous anatomy, computer‐assisted surgery (CAS) was introduced 30 years ago to aid surgeons in reducing surgical errors and ensuring precise prosthesis implantation^15^. In addition, CAS has been shown to improve the accuracy of prosthesis implantation and reduce the proportion of outliers for lower limb MA compared to conventional TKA^16^. The major shortcomings of CAS are a complicated registration process^17^, a steep learning curve, pin‐site‐related complications, and periprosthetic fractures of the tibia and femur^18^. However, a number of researchers argue that better lower limb alignment does not lead to better functional outcome^19^.

To avoid the shortcomings of CAS, navigation devices have been developed that use accelerometer‐based electronic components^20^, ^21^. They combine the alignment accuracy of CAS systems with the familiarity of conventional techniques while avoiding the preoperative imaging and large computer console required for registration and alignment feedback in the process of operating CAS^22^. The utility of the technique remains controversial, and its cost effectiveness remains unclear.

The iASSIST (Zimmer, Warsaw, IN, US) navigation device is a handheld computer‐assisted accelerometer‐based stereotaxic system that simplifies the registration process^23^. The iASSIST system is validated in cadavers and clinical studies by Scuderi *et al*.^24^, who use this unique technique to resolve issues of lower limb alignment reconstruction. To reduce operational complexity, the accelerometer, gyroscope, and wireless communication system are integrated into a small pod which attaches to the femoral and tibial resection jigs. After several simple registration steps, the pods display alignment information for the surgical field without altering the surgical steps, guiding femoral and tibial resection and providing the surgeon with the opportunity to verify bone resection steps and make further adjustments as necessary.

In the past 3 years, studies on the iASSIST system have gradually increased in number, with some researchers believing that it may improve the accuracy of lower limb alignment^25^, ^26^, ^27^, ^28^, ^29^, ^30^, although some have found no significant difference between the iASSIST and conventional techniques^22^, ^23^. The purpose of this meta‐analysis is to determine whether the iASSIST navigation system contributes to more accurate lower limb alignment, improving postoperative functional outcome, compared with conventional surgical techniques.

## Materials and Methods

### *Study Selection*

We conducted a comprehensive electronic search, in PubMed, Embase, Cochrane Central Register of Controlled Trials, Web of Science and CNKI databases, of literature published between 1 January 2000 and 1 October 2018, using the following terms: “iASSIST”, “Zimmer iASSIST”, “TKA”, and “MA.” This study was based on the Cochrane Review Methods, and reporting was carried out according to the Preferred Reporting Items for Systematic Reviews and Meta‐analyses (PRISMA). The review protocol has been registered in the International Prospective register of systematic reviews [CRD42019128880].

Both Chinese and English publications were included. Relevant studies were identified by reading the titles and abstracts of the manuscripts. If it was considered that the primary selection had yielded insufficient information, the full text was checked to confirm whether or not to include the article. Any disagreement was solved by discussion.

### *Eligibility Criteria*

Studies were included if the following inclusion criteria were met:

1. The experimental group used iASSIST navigation in primary TKA.
2. The control group consisted of conventional primary TKA.
3. At least one outcome in our meta‐analysis was reported.
4. No infections were reported during the period of observation.
5. The studies were randomized controlled trials (RCT) or prospective and retrospective nonrandomized controlled trials (nRCT).

A study was excluded if any of the following applied:

1. It duplicated data from a study that was already included.
2. It did not stratify analysis between primary and revision TKA.
3. It was a systematic review.

### *Study Quality Assessment*

All studies that were identified as satisfying these criteria were included in this meta‐analysis and independently evaluated by two reviewers. As recommended by the *Cochrane Handbook for Systematic Reviews of Interventions*, the Newcastle–Ottawa Scale (NOS; using the range 0–9)^31^ was used to assess studies that were included. The NOS evaluates the risk of bias for each study using three criteria: (i) selection of the exposed and unexposed study populations; (ii) comparability; and (iii) outcome measures. The maximum scores for these three factors are 4 stars, 2 stars, and 3 stars, respectively. Studies with a total score ≥7 were considered high quality; those scoring 6 were considered moderate quality and those that scored less than 6 stars were considered low quality. Any disagreements in the bias assessment were resolved by discussion.

### *Data Extraction*

Two reviewers independently extracted data from each study included in the review. Extracted data comprised: first authorʼs name, publication year, study type, sample size, duration of follow up, measured parameters, and the NOS score. Patient information extracted from the studies included: age, gender, and body mass index (BMI). Primary outcomes were postoperative MA angle, having an ideal value of 0°, and outliers from the MA. The coronal angle of the femoral (CFA) or tibial component (CTA) in relation to the MA were regarded as secondary radiographic outcomes, with an ideal value of 90°. Duration of surgery was collected as a tertiary outcome. Knee Society Score (KSS) was used to assess short‐term clinical outcome. Data were extracted independently by two reviewers, with discrepancies in the extracted data resolved by discussion.

### *Statistical Methods*

This meta‐analysis was performed using Cochrane Collaboration Review Manager 5.3 software. The mean difference (*MD*) and its corresponding 95% confidence interval (*CI*) for each measure were calculated for continuous measures; namely, the angles measured in each study. The odds ratio (*OR*) and its corresponding 95% *CI* were calculated for dichotomous measures, “yes” or “no” being the sole options. Heterogeneity was assessed using the *I* ^2^ statistic, the proportion of variation across studies occurring not as a result of chance. Significant heterogeneity was established for *I* ^2^ values >50% and *P* < 0.05. A fixed‐effects model was used for outcome data without significant heterogeneity; for those with significant heterogeneity, a random‐effects model was used. Values of *P* < 0.05 were considered statistically significant. In addition, where heterogeneity was significant, a sensitivity analysis was conducted by omitting studies individually in turn.

## Results

### *Search*

**Figure 1:**
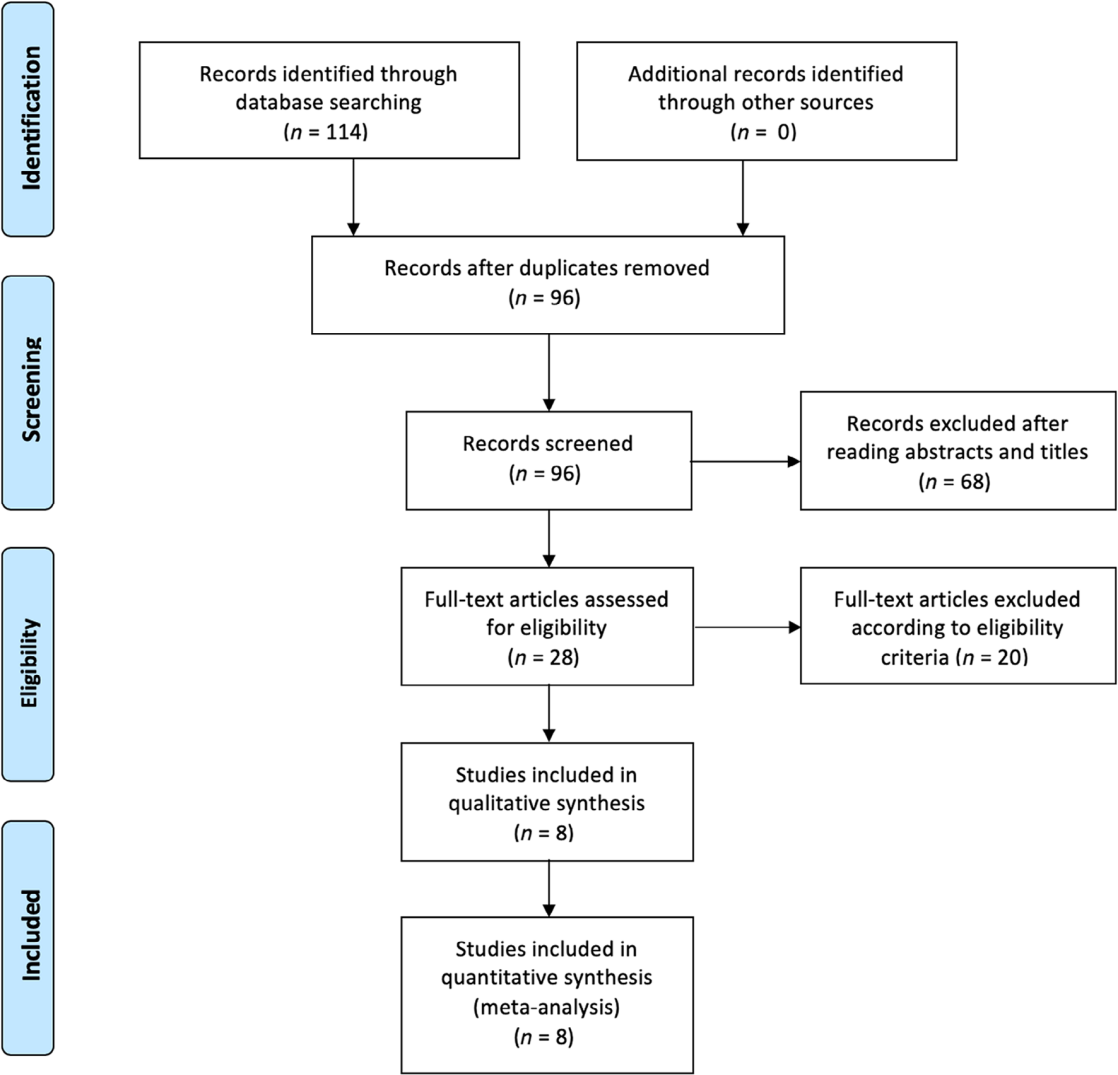
Flow chart.

Figure 1 is a flow chart of the included and excluded studies. A total of 114 articles were identified for the meta‐analysis. Of these, 96 studies were duplications and 68 studies were excluded because the titles or abstracts did not meet the eligibility criteria. After reading full texts, 19 studies were excluded according to eligibility criteria. Finally, 8 studies were included in this review^22^, ^23^, ^25^, ^26^, ^27^, ^28^, ^29^, ^30^, of which 6 studies were in English and 2 in Chinese language.

### *Characteristics*

The characteristics of each study included in this review are outlined in Table 1. A total of 5 RCT and 3 retrospective studies reported a total of 558 knee cases, of which 275 patients used the iASSIST navigation system and 283 underwent conventional surgery. All these studies used iASSIST navigation as navigation devices; 7 studies performed standard invasive TKA and 1 study conducted minimally invasive TKA. The average age of patients from the iASSIST and conventional groups was 66.5 and 66.4, respectively. The average BMI of patients from both groups was 27.1 and 27.0, respectively. The percentage of female patients from both groups was 70.2% and 71.7%, respectively. There were no significant differences between these two groups in terms of age, BMI, and gender. Of these, 8 studies evaluating mechanical alignment, the occurrence of malalignment (13.3%) in the conventional group was better than that (29.04%) in the iASSIST group in 3 studies, but no differences between these two groups in the other 5 studies were observed. Moreover, 3 articles reported that there were no significant differences in postoperative blood loss‐related indicators between these two groups; 2 articles reported that there was no significant difference in postoperative patient satisfaction between these two groups. All original data have been uploaded to the Open Science Framework (osf.io).

**Table 1:** Characteristics of included studies

Author	Year	Study design	iASSIST	CONV	Follow up	Results			*P‐*value
							iASSIST	CONV	
Satit *et al*.	2016	Mechanical axis (MA), Placement of components Surgical time	40	40	6 weeks postoperative	MA (°) MA outliers CFA (°) CTA (°) Surgical time (min)	180.8 ± 2.1 3 90.3 ± 1.0 90.5 ± 1.8 96 ± 14.2	179.9 ± 3.2 10 90.7 ± 2.2 89.3 ± 1.9 94 ± 18.7	0.141 0.031 0.303 0.005 0.65
Tian *et al*.	2017	MA, Placement of components KSS, VAS, surgical time	20	20	Immediate for radiological, 1 month for KSS and VAS	MA shifting (°) CFA shifting (°) CTA shifting (°) Surgical time (min) KSS	1.1 ± 1.1 0.9 ± 0.7 1.2 ± 0.9 80.9 ± 7.8 77.1 ± 8.6	2.9 ± 2.8 2.4 ± 0.9 2.2 ± 1.6 73.1 ± 9.1 70.2 ± 8.1	0.01 <0.01 <0.01 <0.01 <0.05
Wei *et al*.	2018	MA, Placement of components KSS, Hb loss, surgical time	12	12	3 months postoperative for radiological and KSS	MA shifting (°) CFA shifting (°) CTA shifting (°) Surgical time (min) KSS Hb loss (g/L)	1.2 ± 0.45 0.86 ± 0.19 1.06 ± 0.26 67.4 ± 4.99 85.83 ± 4.3 10.42 ± 2.02	2.33 ± 0.62 2.01 ± 0.47 1.326 ± 0.23 63.3 ± 5.01 80.08 ± 5.18 19.08 ± 2.778	0.001 0.001 0.016 0.058 0.007 0.001
Denti *et al*.	2018	MA Tibial slope Range of motion Occasional pain	10	10	Immediate for radiological, 1 year for range of motion and occasional pain	MA shifting (°) Tibial slope (°) Full ROM Occasional pain	2.44 ± 2.01 1.94 ± 1.50 9 1	2.03 ± 1.15 3.04 ± 2.00 8 1	>0.05 >0.05 >0.05 >0.05
Kinney *et al*.	2018	MA, placement of components tourniquet time	25	25	1 and 4 months postoperative	MA shifting (°) CFA shifting (°) CTA shifting (°) Tourniquet time (min)	1.92 ± 0.34 1.65 ± 0.17 1.28 ± 0.13 113.6 ± 2.5	2.83 ± 0.41 2.23 ± 0.33 1.71 ± 0.24 114.3 ± 3.2	0.09 0.12 0.12 0.86
Liow *et al*.	2016	MA, placement of component, KSS, OKS, SF‐36	92	100	1 months for radiological; 6 months for KSS, OKS, and SF‐36	MA shifting (°) MA outliers CFA shifting (°) CTA shifting (°) KSS Surgical time (min)	1.9 ± 1.4 8.7 1.6 ± 1.3 1.6 ± 1.2 71.7 ± 16.6 83.9 ± 21.8	2.8 ± 2.0 26 2.1 ± 1.5 2.1 ± 1.5 69.9 ± 16.4 72.5 ± 14.6	0.001 0.001 0.024 0.024 0.110 <0.001
Moo *et al*.	2018	MA, placement of component, surgical time	30	30	Immediate postoperative	MA (°) MA outliers (%) CFA (°) CTA (°) Surgical time (min)	176.75 ± 0.75 43 91.75 ± 0.75 91.25 ± 0.75 96.25 ± 6.25	176.75 ± 0.75 36 92 ± 0.5 90 ± 0.5 90.62 ± 4.37	0.332 0.384 0.453 0.28 0.13
Lo *et al*.	2018	MA, placement of component, surgical time	46	46	6 months postoperative	MA shifting (°) MA outliers CFA shifting (°) CTA shifting (°) Surgical time (min)	0.19 ± 2.06 3 0.37 ± 1.36 0.24 ± 1.43 96 ± 14.2	0.69 ± 3.18 10 0.90 ± 2.21 0.25 ± 2.62 94 ± 18.7	0.372 0.045 0.172 0.972 0.613

The lower extremity mechanical axis (MA) is the angle formed by the MA of the femur (line between the center of the femoral head and the center of the knee) and the MA of the tibia (line between the center of the talus and the center of the knee or hip‐knee‐ankle angle); the coronal femoral‐component angle (CFA) is the angle formed by the femoral component and the MA of the femur; coronal tibia‐component angle (CTA) is the angle formed by the tibia base plate and the MA of the tibia; the accepted values used in study for normal alignment were: 3° varus/valgus for MA,the outlier were defined as cases in which the alignment error was >3° from the accepted values; KSS, Knee Society Score; VAS, visual analogue scale.

### *Quality*

The methodological quality of each included study was assessed in accordance with the NOS. The NOS score of included studies ranged from 6 to 9, with an average score of 7.12. A total of 5 studies were high quality^23^, ^25^, ^27^, ^29^, ^30^, and the other 3 were moderate quality studies^22^, ^26^, ^28^.

### *Mechanical axis*

**Figure 2:**
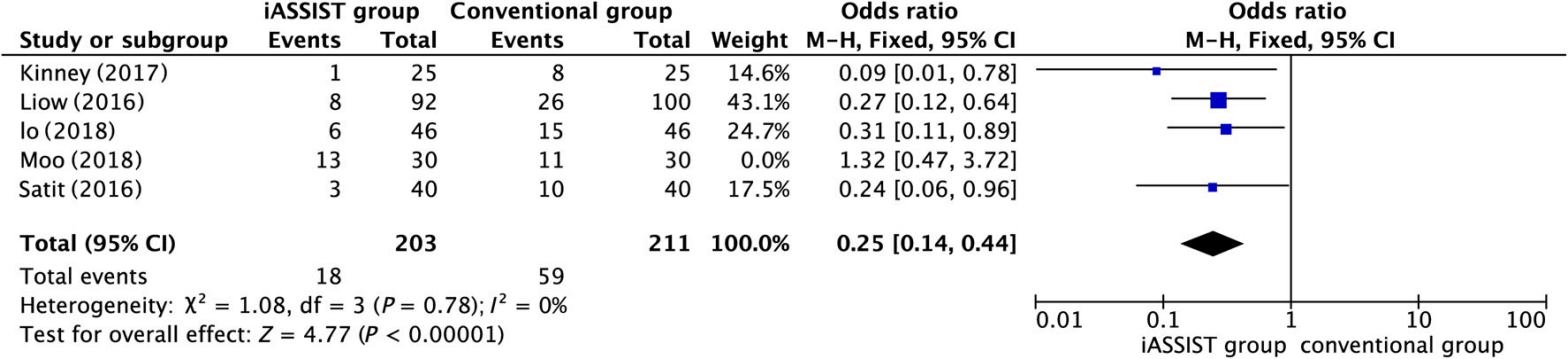
Postoperative mechanical axis (MA) outliers.

**Figure 3:**
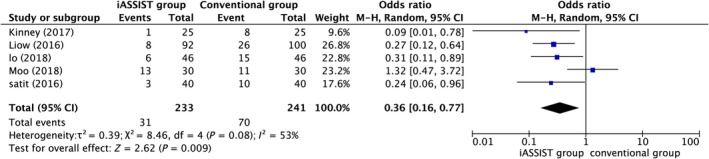
After omission of postoperative mechanical axis (MA) outliers.

5 studies provided MA outlier data, which indicated that there was a significant difference between the iASSIST and conventional groups (*P* = 0.009), with iASSIST reducing the proportion of such outliers (Fig. 2). The value of *I* ^2^ was >50%, signifying significant heterogeneity. The omission of 1 study^22^ changed the value of *I* ^2^ to 19%, suggesting that this article was the source of heterogeneity (Fig. 3).

**Figure 4:**
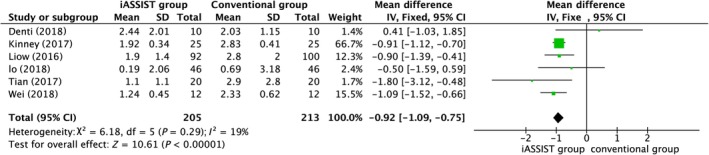
Postoperative mechanical axis (MA).

Six studies provided data on MA, analysis of which demonstrated that highly significant differences were found between the iASSIST and conventional groups (*P* < 0.00001), indicating that iASSIST can significantly improve the accuracy of lower limb alignment compared to conventional treatment (Fig. 4).

### *Coronal Prothesis Angle*

**Figure 5:**
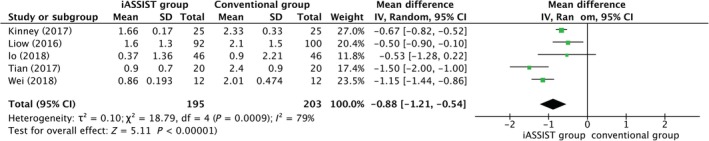
Postoperative coronal femoral angle (CFA).

**Figure 6:**
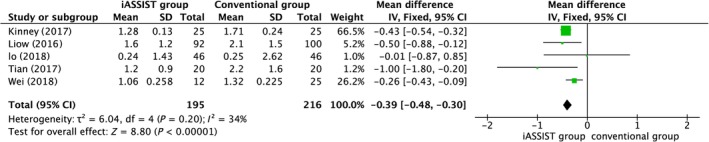
Postoperative coronal tibial angle (CTA).

Five articles measured the coronal femoral angle (CFA) and the coronal tibial angle (CTA). The meta‐analysis indicated that for both CFA and CTA, the iASSIST group was significantly different from the conventional group (*P* < 0.00001), indicating that iASSIST improved the accuracy of prosthesis placement of both components in the coronal plane (Figs. 5 and 6). However, with *I* ^2^ = 79%, the heterogeneity was considered significant for CFA. This value did not change greatly as articles were omitted in turn; thus, this result can be regarded as stable.

### *Operation Time*

**Figure 7:**
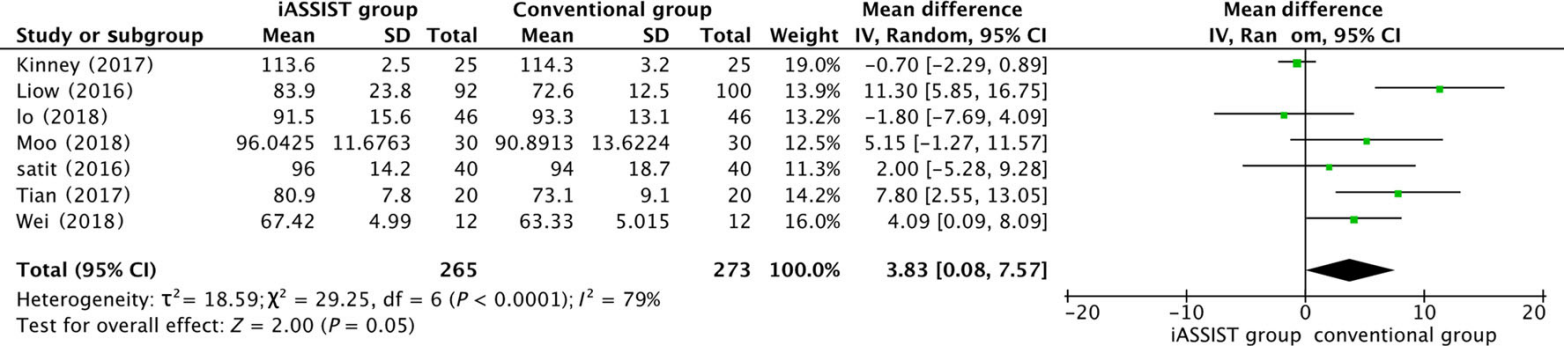
Duration of surgery.

Seven articles discussed duration of surgery. The mean duration of procedures using iASSIST compared to conventional surgery was 90.24 min versus 85.01 min, indicating that iASSIST was a significantly longer process (Fig. 7).

### *Clinical Outcome*

**Figure 8:**
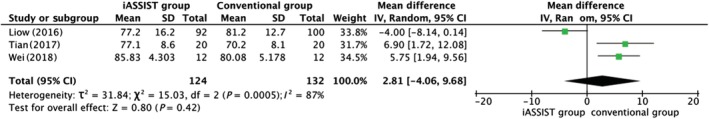
Postoperative Knee Society Score (KSS).

**Figure 9:**
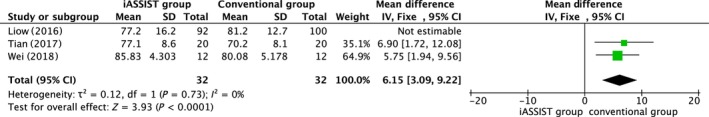
After omission of postoperative Knee Society Score (KSS).

Postoperative KSS, a short‐term outcome measure, was featured in only 3 articles. The meta‐analysis indicated that there was no significant difference between iASSIST and conventional surgery in this regard (*P* = 0.86) (Fig. 8). Heterogeneity was significant. Omission of Liowʼs article^27^ resulted in the *I* ^2^ value falling to 0%, suggesting that this article was the source of heterogeneity (Fig. 9).

## Discussion

Total knee arthroplasty has proven to be a successful procedure, with approximately 250,000 operations performed in China annually, although TKA is not as satisfactory as total hip arthroplasty (THA). Approximately 20% of patients who have undergone TKA have been dissatisfied with their surgery, believed to be because of the surgeonʼs surgical technique. Conventional surgery relies on an intra‐medullary guiding system for cutting bones. Some factors may influence the application of such a system, including varus or valgus deformity, obesity, or a narrow canal. In addition, intra‐marrow penetration will lead to increased bleeding, leading to decreased hemoglobin levels and an increased risk of requiring blood transfusion. Clinicians have considered, therefore, various approaches to achieve a more satisfactory outcome, such as CAS or patient‐matched instrumentation (PMI). Previous studies found that CAS decreased the risk of malalignment to achieve superior short‐term functional outcome^32^. CAS has numerous limitations, such as complex landmark registration, pin complications, a steep learning curve, increased duration of surgery, and questionable cost effectiveness^21^, ^33^, ^34^, ^35^. PMI is an additional technique that assists surgeons in achieving improved lower limb alignment, but a 3D CT scan is required before surgery, and once osteotomy has been performed, the correction in alignment cannot be verified^36^. Conversely, the iASSIST system can verify alignment after osteotomy to ensure its accuracy. The authors of a recent study also noted that PMI was not able to reduce the number of outliers^37^. The iASSIST guidance system allows simple registration and displays alignment information for the surgical field without altering the actual surgical steps, thereby avoiding a number of pertinent complications caused by additional pinning, such as pin tract infections and pain. Therefore, the iASSIST system appears to circumvent the shortcomings of CAS and provides a more convenient and quicker surgical procedure^27^.

Results from this meta‐analysis indicate that the iASSIST navigation system offers a significant improvement in both MA and coronal prosthesis implantation compared with conventional techniques (*P* < 0.0001). Other articles comparing accelerator‐based navigation devices have come to similar conclusions, that such devices can improve the accuracy of lower limb alignment and, therefore, prosthesis implantation^38^, ^39^, ^40^. This meta‐analysis found that 89.1% of iASSIST surgeries, compared with 70.96% of conventional treatments, were within 3° of neutral MA, establishing that the iASSIST guidance system provides more accurate prosthesis implantation, which will reduce the risk of prosthesis loosening and early failure of TKA. We found that the studies from China all had positive results, probably because the Chinese patients had a smaller BMI, which results in fewer problems in surgery than in Western countries. However, due to the time required to register and install the navigation components, the duration of surgery of the iASSIST group was greater than conventional surgery. Contrary to some previous studies using CAS, there was no significant difference between the iASSIST system and conventional surgery in short‐term KSS. This may be due to the fact that the iASSIST system does not alter the actual surgical procedure used in conventional TKA to any significant degree as the principle is to provide accurate alignment information during surgery; studies that have much longer‐term follow up are required to establish its benefits.

Although we have presented here a high‐quality review of the iASSIST guidance system and compared it with conventional surgery, there are several limitations to the study. First, significant heterogeneity was observed, possibly because of differences in skill and experience of the various surgeons, different cutting guides in the conventional procedure, and the diversity of characteristics of patients from different countries and study type. In this article, 5 prospective RCT and 3 retrospective studies were included. There are currently 2 RCT underway that compare iASSIST with conventional surgical techniques. In general, conclusions are more convincing if only RCT are selected for a meta‐analysis, but the current state of the published literature suggests that to do so would result in too small a sample size to draw conclusions. In the future, a meta‐analysis including only RCT will provide additional evidence to guide clinical treatment, if necessary. Only coronal prosthesis angles were considered as the outcome measure, with no sagittal prosthesis angles included due to insufficient data. In addition, various studies have different follow‐up times, which may result in inconsistencies in the calculation of KSS. Measurement standards may vary from study to study, with errors in the recorded measurements. Thus, as a result of insufficient data, we were unable to analyze the complication rates, prothesis survival, and long‐term functional outcomes.

Unlike earlier research, recent studies have shown that a reduction in the numbers of MA outliers due to the use of CAS does not lead to better functional outcomes^41^, ^42^. Even the connection between MA and prosthesis survival may be overstated. Despite this debate, a neutral MA remains the “gold standard” for target alignment, supported by considerable quantities of data^43^. Therefore, more long‐term follow‐up studies are required to demonstrate the relationship between better postoperative lower limb alignment with postoperative functional outcome and prosthesis survival. However, high BMI and severe varus and valgus deformity may increase the deviation of lower limb alignment and the number of MA outliers^5^, ^44^, ^45^. Hence, subgroup meta‐analysis will be required to clarify the clinical significance of the iASSIST system when more detailed relevant literature becomes available.

In conclusion, the present systematic review and meta‐analysis demonstrates the superiority of the iASSIST navigation system, establishing that it provides improved alignment in lower limb reconstruction. However, the duration of surgery when using the iASSIST system is longer than for conventional techniques. Superior radiographic results are not associated with enhanced short‐term functional outcomes.
